# Genotoxic and Mutagenic Effects of the *Alternaria* Mycotoxin Alternariol in Combination with the Process Contaminant Acrylamide

**DOI:** 10.3390/toxins15120670

**Published:** 2023-11-24

**Authors:** Francesco Crudo, Chenyifan Hong, Elisabeth Varga, Giorgia Del Favero, Doris Marko

**Affiliations:** 1 Department of Food Chemistry and Toxicology, Faculty of Chemistry, University of Vienna, Währinger Str. 38–40, 1090 Vienna, Austria; francesco.crudo@univie.ac.at (F.C.); chenyifan.hong@hotmail.com (C.H.); elisabeth.varga@vetmeduni.ac.at (E.V.); giorgia.del.favero@univie.ac.at (G.D.F.); 2 Core Facility Multimodal Imaging Faculty of Chemistry, University of Vienna, Währinger Str. 38, 1090 Vienna, Austria

**Keywords:** genotoxicity, mutagenicity, alternariol, acrylamide, *Alternaria* mycotoxins, γH2AX

## Abstract

Humans are constantly exposed to mixtures of different xenobiotics through their diet. One emerging concern is the *Alternaria* mycotoxin alternariol (AOH), which can occur in foods typically contaminated by the process contaminant acrylamide (AA). AA is a byproduct of the Maillard reaction produced in carbohydrate-rich foods during thermal processing. Given the genotoxic properties of AOH and AA as single compounds, as well as their potential co-occurrence in food, this study aimed to assess the cytotoxic, genotoxic, and mutagenic effects of these compounds in combination. Genotoxicity was assessed in HepG2 cells by quantifying the phosphorylation of the histone γ-H2AX, induced as a response to DNA double-strand breaks (DSBs). Mutagenicity was tested in *Salmonella typhimurium* strains TA98 and TA100 by applying the Ames microplate format test. Our results showed the ability of AOH and AA to induce DSBs and increase revertant numbers in *S. typhimurium* TA100, with AOH being more potent than AA. However, no synergistic effects were observed during the combined treatments. Notably, the results of the study suggest that the compounds exert mutagenic effects primarily through base pair substitutions. In summary, the data indicate no immediate cause for concern regarding synergistic health risks associated with the consumption of foods co-contaminated with AOH and AA.

## 1. Introduction

Molds of the genera *Aspergillus*, *Penicillium*, *Fusarium*, and *Alternaria* are known to produce a broad spectrum of structurally different mycotoxins that can contaminate various types of crops and food commodities and induce a variety of toxicological effects [1]. The contamination of food by these fungal toxic metabolites occurs in both developed and developing countries, with the latter generally showing higher contamination levels [2]. However, due to growing globalization and climate change enabling more favorable conditions for the growth of these fungi, an increased occurrence of such toxic metabolites in food is also expected in the near future in developed countries [3].

Among the various mycotoxins known to date, those produced by the genus *Alternaria* are still not regulated due to the shortage of toxicological and occurrence data which prevents a proper risk assessment from being conducted. In fact, unlike other mycotoxins, for which their maximum levels in food have been set by the Commission Regulation (EU) 2023/915 [4], only indicative levels are currently available for certain *Alternaria* mycotoxins in food. Of note, the exceedance of these levels only implies the need to investigate the factors leading to the presence of the mycotoxins or the effects of food processing (Commission Recommendation (EU) 2022/553) [5]. *Alternaria* species produce mixtures of secondary metabolites that can be classified into five groups, namely dibenzo-α-pyrones, perylene quinones, tetramic acid derivatives, *Alternaria alternata* f. sp. *lycopersici* toxins, and miscellaneous structures [1]. In this context, the mycotoxin alternariol (AOH), which belongs to the dibenzo-α-pyrones group, is one of the most studied *Alternaria* mycotoxins. It can be found in a wide variety of foods, including grains, tomato, fruits (and their respective products), sunflower seeds/oil, and fermented beverages like beer and wine [1,6]. It is important to highlight that data on food occurrences consistently identify AOH as one of the most commonly detected *Alternaria* mycotoxins in food, with food often experiencing simultaneous contamination by multiple *Alternaria* mycotoxins [1]. Of note, a previous study suggested that the actual occurrence may even exceed the present estimate [7]. With respect to its toxicological properties, AOH has been extensively reported to exert estrogenic effects [8,9] as well as to induce different types of DNA damage, including single-strand breaks (SSB), double-strand breaks (DSB), and oxidative DNA damage through mechanisms that involve the generation of reactive oxygen species and the poisoning of DNA topoisomerases (TOPOs), especially the IIα isoform [10]. TOPOs are a family of enzymes that regulate the DNA topological state by breaking one (TOPO I) or both (TOPO II) strands of the DNA which are subsequently rejoined. Thus, the mycotoxin AOH is able to stabilize the DNA–topoisomerase complex that is formed during DNA replication or transcription, thus inhibiting the re-ligation of the DNA which results in the persistence of single- (TOPO I poisoning) and double- (TOPO II poisoning) strand breaks [10,11]. AOH was also reported to act as a mutagenic compound, being able to induce hypoxanthine-guanine phosphoribosyltransferase (HPRT) mutations in V79 cells and thymidine kinase (TK) gene mutations in mouse lymphoma cells [12].

TOPO activity has also been reported to be affected by some process contaminants, including acrylamide (AA) [13]. AA is a small hydrophilic compound primarily formed in carbohydrate-rich foods, such as baked products, through the Maillard reaction during thermal processing at high temperatures (equal to or higher than 120 °C) [14,15]. In vivo studies have reported AA to be quickly absorbed from the gastro-intestinal (GI) tract, to be distributed throughout the whole body and extensively metabolized by conjugation with glutathione or epoxidation to glycidamide (GA) [16,17]. The genotoxicity and mutagenic potential of AA and its reactive metabolite GA have been studied extensively both in vitro and in vivo. In particular, an in vitro study found AA to induce the formation of DNA adducts (mostly N7-guanine adducts), which can lead to depurination or mispairings and, therefore, an increased mutation rate during DNA replication [18]. AA itself has been shown to induce chromosomal aberrations, micronuclei, and other mitotic disturbances in mammalian cells [19]. Based on the scientific data available, the International Agency for Research on Cancer (IARC) has classified AA as a “probable human carcinogen” (group 2A) [16]. Similar to AOH, there are currently no established maximum levels in food for AA. However, the Commission Regulation (EU) 2017/2158 provides benchmark levels for AA, which are not safety levels and only serve as performance indicators. Exceeding these benchmark levels triggers food business operators to review mitigation measures [20]. With respect to the ability of AA to target TOPOs, in vitro studies have found AA to act as a catalytic inhibitor of TOPO II in V79 Chinese hamster cells [13], thus acting differently from the mycotoxin AOH. In fact, unlike TOPO poisons, catalytic inhibitors bind to the enzyme and interfere with its ability to cleave and re-ligate DNA strands without stabilizing the protein–DNA cleavage complexes.

Apart from the previously mentioned food commodities, AOH has been found to occur in several thermally processed cereal-based products, such as wheat and rye bread, breakfast cereals, cookies, and biscuits, which are known to often contain AA [21,22,23]. Despite this, cereal-based products are not among the most contaminated commodities by AOH. Indeed, the concentrations and the frequency of occurrence of AOH in these products are generally much lower than those found in other foods [1]. Nevertheless, the co-exposure of humans to both contaminants remains highly probable. In the context of a varied diet, the consumption of various foods during a meal could, in fact, expose consumers to both contaminants, even if they are present in different food items.

Building upon this foundation and considering their well-known capacity to induce genotoxic and mutagenic effects [1,6,16], the primary objective of the present study was to explore their potential combined genotoxic and mutagenic effects in vitro, which could potentially arise following the consumption of foods contaminated with these widely present compounds. To achieve this and to assess the impact of metabolic activation on the toxicity of the individual compounds and combinations, the In-Cell Western assay of γ-H2AX (genotoxicity) and the Ames microplate format test (mutagenicity) were performed in the presence and absence of the rat S9 fraction.

## 2. Results

### 2.1. CellTiter-Blue^TM^ Cell Viability Assay

**Figure 1:**
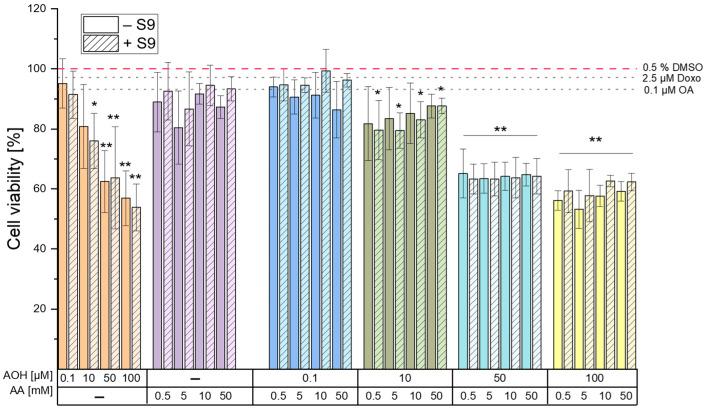
Effects of alternariol (AOH), acrylamide (AA), and their respective binary combinations on viability of HepG2 cells. Cells were incubated with the single compounds/combinations/controls for 4 h in the presence (hatched bar) and absence (solid bars) of rat liver S9 fraction. Okadaic acid (OA) and doxorubicin (Doxo) were used as positive controls in the γH2AX for treatments in presence and absence of metabolic activation, respectively. Results are expressed as mean ± SD of at least 3 biological replicates (compared to the solvent control; 0.5% DMSO). Significant differences between the treatments and the solvent control were assessed by applying the one-way ANOVA with Fisher’s LSD post-hoc test (* *p* < 0.05 and ** *p* < 0.01).

To assess the cytotoxic properties, a CellTiter-Blue^TM^ (CTB) cell viability assay was conducted in the HepG2 cell line after 4 h of incubation with various concentrations of AOH (0.1, 10, 50, and 100 µM), AA (0.5, 5, 10, and 50 mM), and their respective combinations. Figure 1 reports the results of the CTB assay in the presence and absence of the rat liver S9 fraction. Treatments in the presence of metabolic activation did not result in significant changes in the cytotoxic properties of AOH and AA (and corresponding combinations of these) compared to the treatments without metabolic activation, with some exceptions. In particular, while the exposure of cells to the various concentrations of AA did not result in cytotoxic effects when tested alone, single treatments with the mycotoxin AOH resulted in a significant (*p* < 0.05) dose-dependent decrease in the viability of HepG2 cells both in the presence and absence of metabolic activation. However, while a significant reduction in viability was already observed at concentrations as low as 10 µM in the presence of metabolic activation, significant reductions in the absence of the S9 fraction were observed starting from 50 µM AOH. Of note, the most substantial reductions occurred when the cells were exposed to 100 µM AOH, resulting in a viability of 56.9 ± 9.2% and 53.9 ± 7.8% in the absence and presence of the S9 fraction, respectively.

Even though reductions in cell viability were observed after the co-exposure of cells to AOH and AA, such decreases were found to be comparable to those observed after their exposure to the respective single concentrations of AOH, thus suggesting an AOH-driven toxicity without the occurrence of combinatory effects.

The DNA-damaging compounds okadaic acid (OA; 0.1 µM) and doxorubicin (Doxo; 2.5 µM), which were used as positive controls in the γH2AX assay for treatments with and without the S9 fraction (respectively), were also tested for their cytotoxic effects. The CTB results showed no significant cytotoxicity at the tested concentrations (Figure 1).

### 2.2. In-Cell Western Assay of γH2AX (γH2AX Assay)

**Figure 2:**
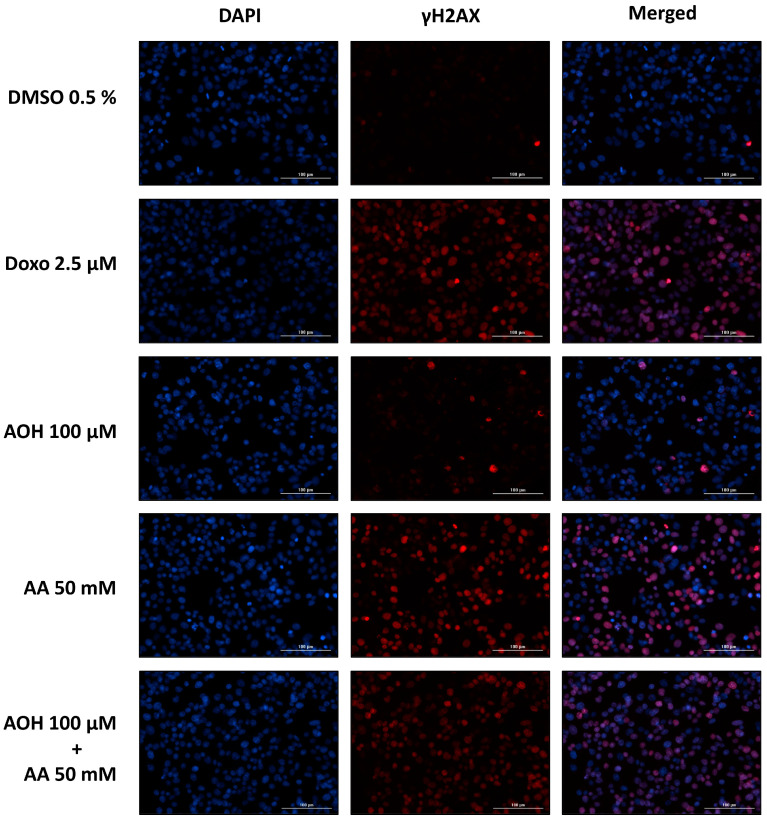
Representative pictures of HepG2 cells exposed to alternariol (AOH) and acrylamide (AA) as single compounds and in combination (in the absence of metabolic activation). Doxorubicin (Doxo) and DMSO were used as positive and solvent controls, respectively. Pictures were acquired with an automated fluorescence microscope (4× magnification) after 4 h incubations. Nuclei were stained with DAPI (blue channel), while fluorescent-labeled secondary antibodies (Alexa Fluor 633) were used for the staining of γH2AX (red channel).

The genotoxic effects exerted by AOH, AA, and their respective combinations on HepG2 cells were assessed by conducting a γH2AX assay. In particular, the same incubation conditions used to assess the impact of the food contaminants on cell viability were applied for the genotoxicity assessment (see Section 2.1). Representative pictures of cells exposed to the controls (0.5% DMSO and 2.5 µM Doxo) and to some selected single and combined treatments (100 µM AOH, 50 mM AA, 100 µM AOH + 50 mM AA) in the absence of metabolic activation are shown in Figure 2.

**Figure 3:**
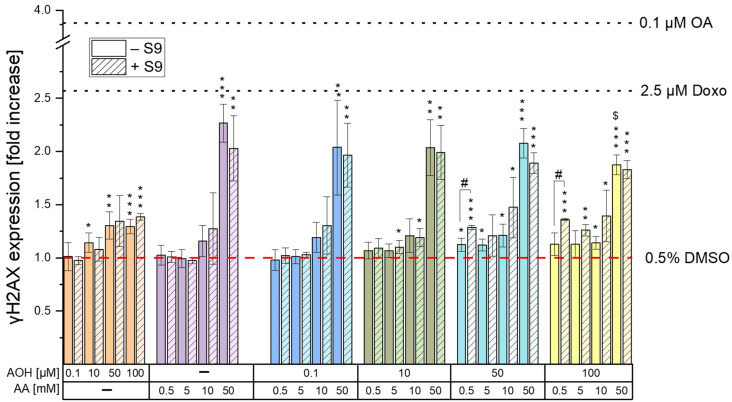
γH2AX expression induced by AOH and AA as single compounds and in combination. HepG2 cells were exposed to the various conditions for 4 h (in presence and absence of metabolic activation). Results are shown as fold increases over the solvent control (0.5% DMSO). Each condition was tested with at least three technical and three biological replicates. Okadaic acid (0.1 µM) and Doxo (2.5 µM) were used as positive controls for treatments with and without S9 fraction, respectively. One-way ANOVA with Fisher’s LSD post-hoc test was applied to determine statistical differences between the various treatments and the solvent control, with * *p* < 0.05, ** *p* < 0.01, and *** *p* < 0.001. Significant differences between single and combined treatments, as well as between treatments with and without metabolic activation were evaluated by applying the Student’s *t*-test. $ indicates a significant difference (*p* < 0.05) between the co-treatments and the respective concentration of AA, while # indicates a significant difference (*p* < 0.05) between the treatments with and without metabolic activation.

Our results obtained for all the single/combined treatments and controls are reported in Figure 3 and expressed as fold increases over the 0.5% DMSO control. As shown in Figure 3, the exposure of the cells to AOH resulted in a significant increase in γH2AX formation, already noticeable at concentrations of 10 µM in the absence of the S9 fraction (1.14 ± 0.09; *p* < 0.05). However, the exposure of cells to higher concentrations of the mycotoxin only slightly increased the γH2AX fluorescence signal, which reached a plateau in the concentration range of 50–100 µM (1.31 ± 0.13 and 1.30 ± 0.07, respectively). Even though similar results were obtained in the presence of metabolic activation, a significant increase in γH2AX expression was only observed at concentrations ≥50 µM. The exposure of cells to AA led to a significant increase in γH2AX formation only at the highest concentration tested (50 mM), with fold increases of 2.03 ± 0.31 (with the S9 fraction) and 2.27 ± 0.18 (without the S9 fraction). Levels of fluorescence comparable to those recorded after the exposure of the cells to 50 mM AA were detected following the treatment of the cells with binary combinations of 50 mM AA and various concentrations of AOH. However, the exposure of the cells to the combination of “50 mM AA + 100 µM AOH” resulted in a significant reduction in γH2AX expression (*p* < 0.001) compared to that of the single treatment with 50 mM AA. Regarding the impact of metabolic activation on γH2AX expression, no significant differences were observed between the treatments with and without the S9 fraction, except for the binary combinations of 0.5 mM AA with 50 and 100 µM AOH, which resulted in an increased γH2AX formation in the presence of the liver S9 fraction.

### 2.3. Ames Microplate Format (MPF) Test

**Figure 4:**
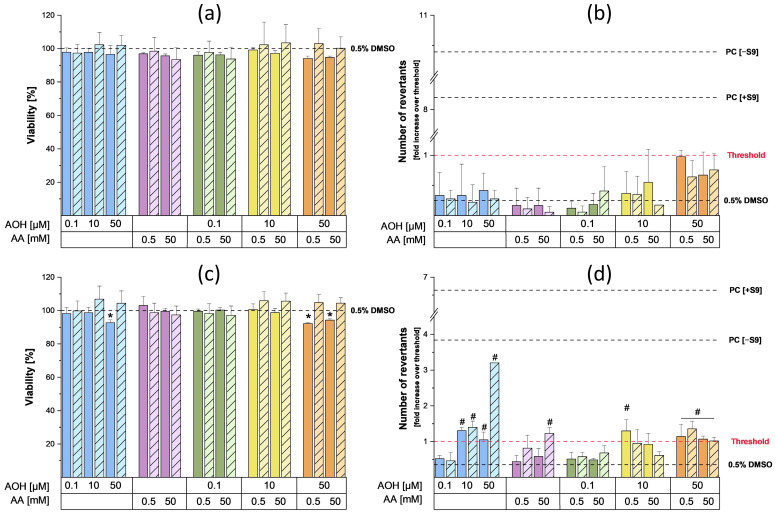
Cytotoxic and mutagenic effects exerted by AOH, AA, and their binary combinations on the *Salmonella typhimurium* strains TA98 and TA100 in the presence and absence of metabolic activation. (**a**,**b**) report the viability and mutagenicity results (respectively) obtained from the exposure of TA98 strain to the test compounds and their combinations for 90 min. (**c**,**d**) report the viability and mutagenicity results obtained in the TA100 strain. Viability results are expressed as mean value (+SD) of the optical densities of three biological replicates, and results were normalized to the solvent control (0.5% DMSO). The * symbol indicates a significant reduction (*p* < 0.05) in bacteria viability compared to the solvent control (0.5% DMSO). Mutagenicity results are expressed as fold increases over the threshold, which were calculated as two times the sum of the mean value of the solvent control plus one standard deviation. The test was considered positive when fold induction was higher than the threshold (as indicated by the # symbol).

The mutagenic effects of AOH (0.1, 10, and 50 µM), AA (0.5 and 50 mM), and their binary combinations were assessed by applying the Ames MPF test, a variation of the traditional Ames test that utilizes liquid media in a high-throughput screening setup. Before performing the Ames test, the cytotoxic effects of the compounds/their combinations on the *Salmonella typhimurium* strains “TA98” and “TA100” were assessed by comparing the optical densities at 600 nm of bacterial suspensions exposed for 90 min to 0.5% DMSO (solvent control, SC) with those obtained from their exposure to the various test conditions. The results concerning the mutagenic and cytotoxic effects exerted by the test compounds and their combinations (in the presence and absence of metabolic activation) are reported in Figure 4.

In particular, the exposure of the TA98 strain to the various test conditions did not result in any cytotoxic (Figure 4a) or mutagenic (Figure 4b) effects, both with and without metabolic activation. On the contrary, the treatment of the TA100 strain with 50 µM AOH resulted in a slight but significant reduction in cell viability (viability: 92.7 ± 1.7%) in the absence of the S9 fraction (Figure 4c). While a similar reduction in viability was also observed after the co-treatment of cells with 0.5/50 mM AA and 50 µM AOH, no changes in cell viability were observed following the exposure of the TA100 strain to the other test conditions. Regarding the mutagenic effects, a concentration-dependent increase in the number of revertants was observed for AOH in the presence of metabolic activation, starting at a concentration of 10 µM (Figure 4d). Of note, the exposure of the TA100 strain to the highest concentration of AOH tested (50 µM) resulted in a 3.2-fold increase over the threshold in the number of revertants. In the absence of metabolic activation, the exposure of the TA100 strain to 10 and 50 µM AOH resulted in a non-concentration dependent increase in the number of revertants (with fold increases over the threshold of 1.3 ± 0.2 at 10 µM and 1.0 ± 0.1 at 50 µM). Unlike AOH, exposure to AA resulted in slight mutagenic effects only at the highest concentration tested (50 mM) in the presence of the S9 fraction (with a fold induction of 1.2 ± 0.2). The co-exposure of the TA100 strain to 0.1 µM AOH and the two concentrations of AA (0.5 and 50 mM) did not result in any mutagenic effects, both in the presence and absence of metabolic activation. In the absence of metabolic activation, the combination “10 µM AOH + 0.5 mM AA” resulted in a number of revertants comparable to those found after the treatment with 10 µM AOH alone. However, a significant reduction in the number of revertants was observed following the incubation of bacteria with the combination of 10 µM AOH/50 mM AA compared to that of the treatment with 10 µM AOH alone. No mutagenic effect was observed during combinations of 10 µM AOH with the two concentrations of AA in the presence of metabolic activation. The exposure of bacteria to binary combinations of 50 µM AOH and 0.5/50 mM AA resulted in slight mutagenic effects both in the presence and absence of the S9 fraction. However, in the presence of metabolic activation, a significant decrease in the number of revertants was observed after exposure to the combination of 50 µM AOH/50 mM AA compared to that of the single treatment with 50 µM AOH (Figure 4d). The increased number of revertants observed after the exposure of the TA98 and TA100 strains to the positive controls confirmed the validity of the results obtained.

## 3. Discussion

The emerging *Alternaria* mycotoxin AOH has gained increasing interest from the scientific community over the past few decades due to its high occurrence in food and broad spectrum of adverse effects reported in vitro and in vivo [6]. However, since data currently available are still not sufficient to conduct a comprehensive risk assessment, no regulations are currently in place. AOH was reported to occur in several cereal-based foods, including bakery products [21,22]. These products are known to be often contaminated by the process contaminant AA, whose production occurs at a high temperature of cooking because of the Maillard reaction [24]. Despite the co-occurrence of AOH and AA in food and their well-known individual genotoxic and mutagenic properties [1,6,16], there is currently a lack of knowledge regarding the potential toxicological outcomes on human health associated with their combination. Therefore, the aim of the present study was to assess, for the first time, the possible onset of combinatory genotoxic and mutagenic effects resulting from the co-exposure to these compounds. In addition, the possible occurrence of combinatory cytotoxic effects was also investigated.

The results of the cell viability assay deriving from a 4 h exposure of HepG2 cells to different concentrations of AOH and AA (as single compounds or in combination) revealed the inability of AA to induce cytotoxic effects up to the highest concentration tested (50 mM). On the contrary, AOH was found to decrease the viability of HepG2 cells in a concentration-dependent manner (Figure 1) starting from a concentration of 10 µM (in the presence of metabolic activation). The exposure of cells to 100 µM AOH resulted in viabilities of 53.9 ± 7.8% and 56.9 ± 9.2% in the presence and absence of metabolic activation, respectively. Of note, a decrease in the viability of HepG2 cells to 53 ± 13% was also reported by Hessel-Pras et al. [25] after the exposure of the cells to 100 µM AOH for the same incubation time. Furthermore, Vejdovszky et al. [26] showed the ability of AOH to induce a dose-dependent cytotoxic effect in the HepG2 cell line (EC50: 51.4 µM), with no further significant decrease in cell viability at concentrations higher than 50 µM. These results are in accordance with those obtained in the present study, in which the exposure of cells to 50 µM and 100 µM AOH resulted in a similar cell viability (62 ± 10.3% and 56.9 ± 9.2%, respectively). As shown in Figure 1, the treatment of cells with binary combinations of AOH and AA resulted in decreased cell viability without any observable combinatory effects. In fact, the changes in cell viability almost completely overlapped with those induced by the respective concentrations of AOH tested individually, thus suggesting AOH-driven cytotoxic effects.

The genotoxic properties of the *Alternaria* mycotoxin AOH in combination with AA were assessed by quantifying the phosphorylated histone γ-H2AX, whose production occurs following the induction of DNA double-stand breaks [27]. As shown in Figure 3, the exposure of cells to 0.5–50 mM AA resulted in a significant increase in γH2AX expression only at the highest concentration tested (50 mM), without differences between treatments with and without the liver S9 fraction. The induction of genotoxic effects by AA on HepG2 cells was previously reported by Jiang et al. [28], who showed an increase in tail length and tail moment in the alkaline comet assay at concentrations far lower (2.5–20 mM) than those found to induce genotoxic effects in the present study (50 mM). This might be attributed to the fact that the alkaline comet assay assesses cumulative DNA damage resulting from both single-strand breaks (SSBs) and double-strand breaks (DSBs), while the phosphorylation of the 2AX histone occurs only in the presence of DSBs [27]. It is important to note that DSBs are ordinarily present in smaller quantities than SSBs [29], which further explains the onset in the present study of genotoxic effects at concentrations higher than those reported in the literature. Unlike AA, the exposure of cells to AOH resulted in a significant increase in γH2AX expression starting from a concentration of 10 µM. However, the increase was only slight and reached a plateau in the concentration range of 50–100 µM. The lack of a further increase in γH2AX expression in this concentration range is unlikely to be a consequence of a lower number of living cells due to cytotoxicity, since the γH2AX expression was assessed by quantifying the mean fluorescence signal per cell nucleus (and not per well). Furthermore, the co-exposure of cells to binary combinations of 50/100 µM AOH with 50 mM AA resulted in an increased γH2AX expression compared to that of the respective treatments with AOH alone. This confirms the non-involvement of the cytotoxic effects induced by AOH in the scarce increase in γH2AX expression during the single treatments with the mycotoxin. A possible explanation for this phenomenon might be the inhibition by the mycotoxin AOH of the kinases involved in the phosphorylation of the 2AX histone. In fact, AOH has been previously reported to inhibit casein kinase 2, probably by interacting with the ATP binding site of the enzyme [30]. Due to the high conservation of ATP binding sites among kinases [31], there is a plausible chance that AOH might also target the kinases involved in γH2AX formation. While additional focused investigations are essential to validate this assumption, this prospective mechanism could also explain the observed decrease in γH2AX expression observed when cells were exposed to the combination of “100 µM AOH + 50 mM AA” as opposed to 50 mM AA alone. In fact, on the one hand, the results from the γH2AX assay suggest a reduction in the genotoxic effects when cells are co-exposed to AOH and AA; however, on the other hand, this contradicts our expectations, given that both compounds individually induce genotoxic effects. Apart from the combination with the highest concentration of AOH, which resulted in antagonistic effects, no relevant combinatory effects were observed following the exposure of HepG2 cells to the other test conditions. In fact, the increases in γH2AX expression observed during the various co-treatments were comparable with those induced by the single treatments with the mycotoxin AOH (in co-treatments with 0.5, 5, and 10 mM AA) or the process contaminant AA (in co-treatments with 50 mM AA), depending on the compound showing the strongest genotoxic effects at the specific concentration tested.

To assess the mutagenic properties of AOH and AA as single compounds and in combination, the Ames MPF test was conducted. For this purpose, two different strains of *Salmonella typhimurium*, namely TA98 and TA100, were used to gain information about the type of mutations induced by the test compounds (TA98: frameshifts mutations; TA100: base pair substitutions) [32]. The results of the preliminary cytotoxicity assessment performed on the strains did not show any cytotoxic effects following incubation with the various concentrations of AA. On the contrary, slight cytotoxic effects in the TA100 strain were observed in the presence of metabolic activation during the single and combined treatments with 50 µM AOH (the viability was always above 90%). As shown in Figure 4b, the exposure of TA98 strain to 0.5 and 50 mM AA did not result in any increase in the number of revertants, while a slight but significant increase over the threshold was detected in the TA100 strain following exposure to 50 mM AA in the presence of metabolic activation (Figure 4d). The increase in revertants observed after metabolic activation was probably a consequence of the metabolization of AA into its reactive metabolite glycidamide (GA), which is known to exert mutagenic effects in the *S. typhimurium* TA100 strain [33] and in several eukaryotic cell lines (formation of DNA adducts) [18]. However, the currently available data on the mutagenic properties of AA on *S. typhimurium* strains are often inconsistent. In fact, while Yang et al. showed weak positive results in both strains after metabolic activation [34], other authors did not report any mutagenic effects [35]. According to the IARC and FAO/WHO, the absence of clear mutagenic results in the Ames tests conducted on *S. typhimurium* strains is probably a consequence of the limited availability or complete absence of a particular cytochrome P450 (CYP 450) isozyme (most likely CYP 2E1) in the S9 mix, which is responsible for metabolizing small hydrophilic compounds such as AA [36]. In the present study, the usage of the Ames test in microplate format might also have contributed to our success in showing mutagenic effects in the TA100 strain compared to the previously reported studies in which the standard Ames test was performed. In fact, since the Ames MPF test requires the use of liquid media, bacteria are more in direct contact with the test compounds compared to the classic Ames test, which is performed on agar plates. Regarding the *Alternaria* mycotoxin AOH, the exposure of the TA98 strain to 0.1, 10, and 50 µM AOH did not lead to any increase in the number of revertants compared to the solvent control (Figure 4). On the contrary, mutagenic effects were observed in the TA100 strain starting from 10 µM, both in the presence and absence of metabolic activation. These results are in line with those reported by Schrader et al., who showed the inability of AOH to induce mutagenic effects in the TA98 strain, though it increased the number of TA100 revertants [37]. In the present study, the exposure of the TA100 strain to AOH in the absence of metabolic activity only led to a slight increase in the number of revertants, while a more relevant and concentration-dependent mutagenic effect was observed during treatments in the presence of metabolic activation. To a much lesser extent, an enhancement in the mutagenic properties of AOH in the presence of the S9 fraction was also reported by Schrader et al. [37]. This suggests that the metabolization of AOH could enhance its mutagenic properties. Interestingly, the mutagenic effect exerted by 50 µM AOH in the presence of metabolic activation was decreased by the co-incubation with both concentrations of AA. Even though further dedicated studies are needed to explain this phenomenon, this suppression might be a consequence of the reaction of AOH or AOH metabolites with AA or GA. This reaction could result in a reduced availability of the metabolite(s) responsible for the mutagenic effects induced by the mycotoxin. Finally, the possible activation of detoxification mechanisms by AOH, AA, or their respective metabolites at the concentrations tested cannot be excluded. Apart for the combinations with 50 µM AOH, which suggest the onset of antagonistic effects, no relevant combinatory effects were observed.

In summary, in the light of the results reported above, even though AOH and AA were shown to exert genotoxic and mutagenic effects when tested individually, their combination does not seem to result in the onset of combinatory effects. This was evident even at the concentrations tested, which significantly exceed those typically encountered through dietary exposure in vivo. Indeed, on the one hand, the lowest concentration tested for each of the test compounds might be potentially reached (e.g., at the intestinal level) if the extreme contamination levels reported in the literature are considered within the context of a varied diet; however, on the other hand, more realistic estimated exposure levels at the 95th percentile have been previously reported to range from 0.4 to 2.3 μg/kg bw/day for AA and 4.2 to 54.4 ng/kg bw/day for AOH [16,38]. These exposure levels would result in concentrations much lower than the ones tested in the present study, whose aim was to explore the possibility of the occurrence of combinatory effects in vitro. Nevertheless, it is important to note that, considering the complex composition of food, the potential onset of combined effects due to the co-presence of food bioactive compounds or other contaminants (e.g., mycotoxins from *Alternaria* or other genera) cannot be entirely excluded.

## 4. Conclusions

The present study aimed to investigate the potential combined effects of the emerging *Alternaria* mycotoxin AOH and the process contaminant AA, particularly focusing on genotoxicity and mutagenicity. The results obtained in the HepG2 cell line suggest the absence of synergistic interactions between AOH and AA in terms of genotoxic effects (DSBs), even though both compounds were individually found to induce genotoxic effects. In addition, the results of the study demonstrate the ability of both compounds to induce slight mutagenic effects in the *S. typhimurium* strain TA100, but not in the strain TA98. This suggests that the compounds induce mutagenic effects predominantly through base pair substitutions.

In conclusion, while further comprehensive studies are needed to elucidate the intricate mechanisms underlying the reductions in genotoxic and mutagenic effects observed during the combined treatments involving the highest concentrations of the tested compounds, the data collected support the assertion that there is presently no compelling basis for concern regarding the potential onset of synergistic effects subsequent to the consumption of foods contaminated with these ubiquitous contaminants by consumers. Finally, future investigations should explore the dynamics of DNA repair mechanisms to better characterize the risks deriving from exposure to these food contaminants.

## 5. Materials and Methods

### 5.1. Chemicals

Acrylamide (99%; for molecular biology), alternariol (96%; from *Alternaria* sp.), and doxorubicin hydrochloride were purchased from Sigma-Aldrich (St. Louis, MO, USA), while okadaic acid was acquired from Enzo Biochem Inc. (New York, NY, USA). For cell culture experiments, RPMI (Roswell Park Memorial Institute, Buffalo, NY, USA) 1640 medium, heat-inactivated fetal bovine serum, and penicillin/streptomycin (P/S) solution were purchased from Thermo Fisher Scientific Inc. (Waltham, MA, USA). For the cell viability assay, the CellTiter-Blue^®^ Cell Viability reagent was purchased from Promega Corporation (Fitchburg, MA, USA). The 4′,6-diamidine-2′-phenylindoledihydrochloride (DAPI), Alexa Fluor ™ 633 F(ab’)2 fragment of goat anti-mouse IgG (H+L), bovine serum albumin (BSA; Standard Grade Powder, Fraction V), and S9 fraction (20 mg/mL from liver of Sprague Dawley rats), which were used for the γH2AX assay, were purchased from Thermo Fisher Scientific Inc. (Waltham, MA, USA). Anti-phospho-Histone H2A.X (Ser139) Antibody (clone JBW301) and glucose-6-phosphate di-sodium salt were purchased from Sigma-Aldrich Corporation (St. Louis, MO, USA), while nicotinamide adenine dinucleotide phosphate (NADP) was purchased from Merck KGaA (Darmstadt, Germany).

### 5.2. Cell Culture Conditions

The CTB and γH2AX assays were performed by employing the human hepatocarcinoma HepG2 cell line (European Collection of Authenticated Cell Cultures; Salisbury, UK). Cells were maintained in RPMI 1640 medium supplemented with 10% (*v*/*v*) FBS and 1% penicillin (10,000 Units/mL)/streptomycin (10,000 µg/mL) solution. Cells were grown as a monolayer in a humidified incubator (37 °C, 5% CO_2_), passaged twice per week (70–80% confluency), and routinely tested for mycoplasma contamination.

### 5.3. In-Cell Western Assay of γ-H2AX

To assess the genotoxic properties of AOH (0.1, 10, 50, and 100 µM), AA (0.5, 5, 10, and 50 mM), and their respective binary combinations, the γH2AX assay was performed according to Ebmeyer et al. [39], with some modifications. This assay is based on the quantification of the phosphorylated histone γ-H2AX, whose production occurs following the induction of double-strand breaks. All conditions were tested in the presence and absence of metabolic activation with rat liver S9 fraction. Briefly, 50,000 cells/well were seeded into 96-well plates (black plate, clear bottom; Corning Incorporated, Kennebunk, ME, USA) and allowed to attach for 24 h at 37 °C and 5% CO_2_. Before cell treatment, an S9 mix consisting of 100 mM phosphate buffer (Na_2_HPO_4_/NaH_2_PO_4_), 8 mM MgCl_2_, 33 mM KCl, 4 mM NADP, 5 mM glucose-6-phosphate, and 2 mg protein/mL of the S9 fraction was prepared for incubations requiring metabolic activation. The S9 mix was therefore diluted (1:5) with the various test/control media (RPMI + 1% P/S + controls/test substances) and pre-incubated at 37 °C and 5% CO_2_ for 2 h. After 4 h incubation of cells with the various test conditions (with and without S9), cells were fixed by adding 50 µL of ice-cold methanol per well (20 min at 4 °C). Cells were washed three times with PBS-T (PBS + 0.1% Tween^®^ 20) and blocked (1% BSA in PBS-T) for 1 h at room temperature (subject to shaking). Afterwards, cells were washed once with PBS-T and incubated overnight (4 °C) with 100 µL/well of the primary antibody solution (Anti-phospho-Histone H2A.X (Ser139) Antibody, clone JBW301; 1:400 in blocking solution). Cells were washed three times with PBS-T and incubated for 1 h (at room temperature (RT), in the dark) with 100 µL/well of secondary antibody solution (Alexa Fluor™ 633 F(ab’)2 fragment of goat anti-mouse IgG (H+L); 1:500 in blocking solution). After washing with PBS-T, cell nuclei were stained by adding 50 µL of 3 µM DAPI solution (30 min at RT). Finally, cells were washed three times with PBS-T and plates were stored at 4 °C until the time of analysis. Picture acquisition (two optical fields/well) and quantification of the γH2AX fluorescence signals were performed by using a Lionheart FX automated microscope equipped with the software Gen5 (ver. 3.08; BioTek Instruments Inc., Winooski, VT, USA). The corresponding wavelengths for the detection of the fluorescence signals were 380/460 nm (excitation/emission) for DAPI and 620/655 nm (excitation/emission) for the γ-H2AX. The mean value of the γH2AX signal intensity per cell was calculated and values were normalized to the positive control (2.5 µM doxorubicin). As controls, 0.5% DMSO, 2.5 µM doxorubicin (for treatments without S9 fraction), and 0.1 µM okadaic acid (for treatments with S9 fraction) were used. The final DMSO concentration was always 0.5%. All test conditions were tested in technical triplicates and with 3 independent experiments.

### 5.4. CellTiter-Blue^®^ Assay

The CellTiter-Blue^®^ assay was applied to assess the effects of AOH, AA, and their respective combinations on the viability of HepG2 cells. After the cells were incubated for 4 h with the various test conditions (see Section 5.3 for cell seeding and treatment), the test media were removed and replaced with 100 µL/well of the 1× CTB solution (1:10 dilution of 10× CTB solution with phenol-red free DMEM). After 40 min of incubation at 37 °C and 5% CO_2_, the supernatants were transferred into a black 96-well plate and the fluorescence was measured at 590 nm by means of a microplate reader (Cytation 3 imaging reader; BioTek, USA). All test conditions were tested in technical triplicates and with three independent experiments. Results were expressed as mean fluorescence value of three biological replicates and normalized to the solvent control (0.5% DMSO; set to 100%).

### 5.5. Ames Microplate Format (MPF™) Assay

To assess the mutagenic properties of AOH (0.1, 10, and 50 µM), AA (0.5 and 50 mM), and their respective binary combinations, a commercial Ames kit with aroclor-induced rat liver S9 fraction was employed (Ames MPF 98/100 semisolid kit + Ames MPF S9 Cofactor Kit; Xenometrix^®^ AG, Allschwil, Switzerland). In particular, the test was conducted following the provider’s protocol and by employing the *S. typhimurium* strains TA98 (sensitive to frameshift mutations) and TA100 (base pair substitution). Briefly, overnight cultures of bacteria were used only after reaching an optical density at 600 nm (OD_600_ value) ≥ 2. For incubations without S9 fraction, overnight cultures were diluted to 1:10 (for TA98) or 1:20 (TA100) with exposure media containing the various test chemicals. For incubations with S9 fraction, an S9 mix containing buffer salts, glucose-6-phosphate, NADP, and S9 fraction was prepared according to the provider’s protocol and diluted 1:6 with exposure media containing booster solution (final concentration: 0.16%) and the various test chemicals. For treatments with TA98 and TA100 strains in absence of S9 fraction, a mixture of 2 µg/mL 2-nitrofluorene and 0.1 µg/mL 4-nitroquinoline oxide was used as a positive control. For treatments of TA98 and TA100 in the presence of metabolic activation, 1 and 2.5 µg/mL 2-aminoanthracene were used as positive controls. DMSO (4%) was used as a solvent control (with the same % in all test conditions). Bacterial suspensions (250 µL/well in 24-well plates) were therefore incubated with the various test conditions for 90 min at 37 °C while shaken. At the end of the incubation time, 2.8 mL of indicator medium that lacks histidine was added to each well. Afterwards, 50 µL of each bacterial suspension (treated and diluted in indicator medium) was transferred into 48 wells of a 384-well plate, which was subsequently placed into a resealable plastic bag and incubated for 48 h at 37 °C (without shaking). The plates were scored by counting the wells that turned yellow. Results were expressed as fold increase over threshold, which was calculated as two times the sum of the mean value of the solvent control plus one standard deviation (in accordance with the provider’s instructions). The test was considered positive when the fold induction was higher than the threshold.

To assess the impact of single and combined treatments on the viability of bacteria, a cell viability test (in exposure medium) was performed on bacterial strains exposed to the various test conditions (for 90 min) by measuring the optical density at 600 nm. Furthermore, to avoid misinterpretation of the results due to poor solubility of the compounds, the presence of possible crystals in the wells was assessed by using an inverted microscope.

The concentration range chosen for AOH and AA aligned with OECD guideline n. 471, which delineates the evaluation of the mutagenic properties of chemicals via bacterial reverse mutation tests [40]. Specifically, the highest concentrations tested in this study were those that did not interfere with plate scoring due to precipitation of the compounds.

### 5.6. Statistical Analyses

The statistical analyses were conducted using Origin Pro 2022. Significant differences between single and combined treatments as well as between treatments with and without metabolic activation were evaluated by applying the Student’s *t*-test. One-way ANOVA with Fisher’s LSD post-hoc test was applied to assess the differences between the various treatments and the respective solvent or positive control.

## Data Availability

The data presented in this study are available on request from the corresponding author.
